# SIMPLE: A Novel Scoring System for Predicting Hemodynamically Significant Patent Ductus Arteriosus Without Echocardiographic Evaluation in Extremely Low Birth Weight Infants

**DOI:** 10.3389/fped.2021.649515

**Published:** 2021-03-23

**Authors:** Ilker Gonen, Aslan Babayigit, Helen Bornaun, Beril Yasa, Seyma Memur, Seda Yilmaz Semerci, Merih Cetinkaya

**Affiliations:** ^1^Department of Neonatology, Kanuni Sultan Suleyman Training and Research Hospital, Health Sciences University, Istanbul, Turkey; ^2^Department of Pediatric Cardiology, Kanuni Sultan Suleyman Training and Research Hospital, Health Sciences University, Istanbul, Turkey

**Keywords:** clinical scoring system, echocardigraphy, extremely low birth weight infants, patent ductus arteriosus, SIMPLE

## Abstract

**Aim:** To develop a novel clinical scoring system for predicting hemodynamically significant patent ductus arteriosus (hsPDA) in extremely low birth weight (ELBW) infants.

**Methods:** A prospective observational study was conducted among ELBW infants born in the study center during a 6-month period. Fourteen items were selected on a literature review basis and weighed by severity on an arbitrary 1–4 scale, the sum of which represented the Scoring preterm Infants for PDA cLinically without Echocardiographic evaluation (SIMPLE) score. The SIMPLE scores were compared at several time points during the first 3 days of life between two groups of patients: those with an hsPDA at echocardiography and those without.

**Results:** A total of 48 ELBW infants were enrolled, of which 30 infants developed hsPDA. The SIMPLE scores of the infants with hsPDA were significantly greater than those of the infants who did not develop hsPDA. Cut-off SIMPLE scores that were significantly associated with detection of symptomatic hsPDA at each evaluation time point were identified.

**Conclusions:** SIMPLE is the first scoring system that depends on the risk factors and clinical findings of ELBW infants for early prediction of hsPDA. It is simple, objective and easy to perform, and it does not require any additional tests and/or echocardiographic evaluation. We suggest that SIMPLE can be used as a screening tool for determining the need for echocardiographic evaluation in ELBW infants in order to minimize the number of unnecessary pediatric cardiology consultations.

## Introduction

Ductus arteriosus (DA) is an anatomical channel that exists between the pulmonary artery and the aorta. DA is maintained in open conformation throughout fetal life while its persistency after birth is called patent ductus arteriosus (PDA). Although DA closes physiologically within 72 h of life in term infants, it often remains open due to several factors including immaturity, higher prostaglandin levels, respiratory distress, fluid overload, sepsis, and hypoxia in preterm infants (1–3). Spontaneous PDA closure rates for premature infants <28 weeks and/or 1,000 g was reported as 34 and 41%, on the 3rd and 7th days of life, respectively (4). Since PDA produces a left-to-right shunt, clinical findings of systemic stealing and pulmonary overflow develop after completion of the postnatal transition. Clinical findings of PDA include increased respiratory support and precordial pulses, tachycardia, hepatomegaly, accentuated femoral pulses, oliguria, and metabolic acidosis. PDA is also associated with long-term morbidities, such as bronchopulmonary dysplasia (BPD), necrotizing enterocolitis (NEC), focal intestinal perforation, intraventricular hemorrhage (IVH), retinopathy of prematurity (ROP), neurodevelopmental retardation, and death (3, 5, 6).

Although physical examination, clinical findings, and cardiac biomarkers can be used for diagnosis of PDA, echocardiography (echo) is considered the gold standard as a diagnostic tool (1). The scoring systems that have been developed for the PDA diagnosis in preterm infants depend on echo evaluation, and usually they can be used from the 2nd day of life (7–11). As physical examination needs to be performed at two different time points in one of them, this scoring system also comprises the risk of incompatible results in case two different physicians perform the physical examinations (10). Due to these limitations, the previously reported scoring systems may not be useful for the diagnosis of hemodynamically significant PDA (hsPDA) in early days of life, and therefore, are not widely practiced.

Hence, the aim of this study was to establish an easily applied scoring system that allows rapid, standard, and non-invasive evaluation of hsPDA in the early period of life, by combining both the etiology and clinical findings of prematurity without relying on echo findings in extremely low birth weight (ELBW) infants, since the echo evaluation may be challenging at any time point in many neonatal intensive care units (NICUs).

## Materials and Methods

**Figure 1:**
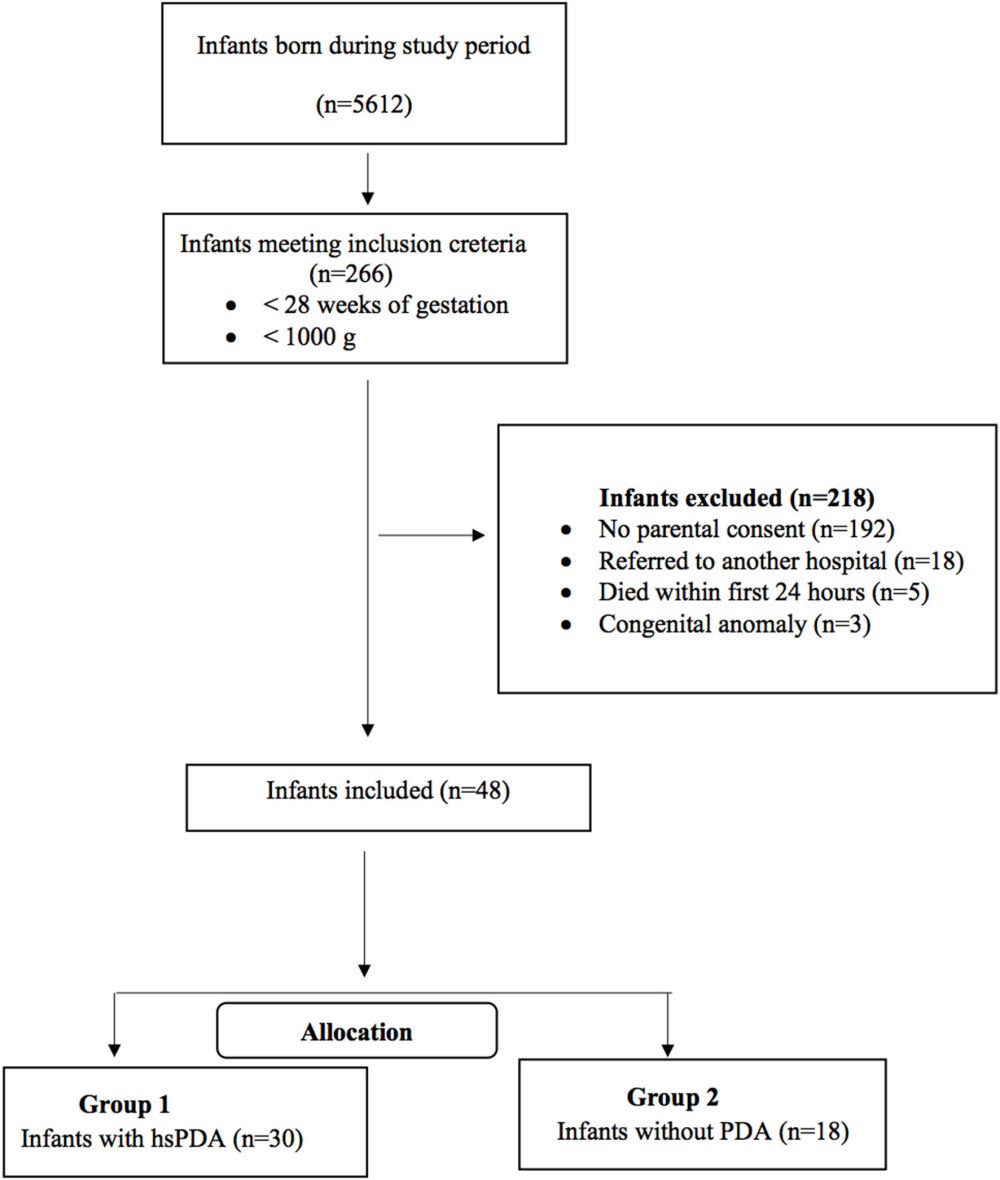
Flow chart of the study population.

This prospective observational study was conducted at Kanuni Sultan Suleyman Training and Research Hospital NICU between November 2019 and April 2020. ELBW infants born at the study center with a gestational age of <28 weeks and/or birth weight <1,000 g were eligible for the study. Infants with major congenital anomalies, those who died within the first 24 h of life, and whose parents did not provide signed informed consents were excluded from the study. We established a new scoring system, “SIMPLE” (Scoring preterm Infants for PDA cLinically without Echocardiographic evaluation) for prediction of hsPDA in ELBW infants. The scores were compared at several time points during the first 3 days of life between two groups of patients: those with an hsPDA at echocardiography and those without. The study was approved by the local ethical committee. The flow chart of the study has been presented in Figure 1.

The SIMPLE score was elaborated using 14 parameters identified on a literature review basis and weighed by severity on an arbitrary 1–4 scale. These items, which represent established risk factors for PDA, include maternal chorioamnionitis, antenatal steroids, birth weight, cord blood gas, hypotension requiring inotropic support, heart rate, invasive mechanical ventilation (MV) requirement, metabolic acidosis, respiratory acidosis, maximum peak inspiratory pressure (PIP), reduction of PIP, fraction of inspired oxygen (FiO_2_) upon admission, FiO_2_ decrease over evaluation time points, and surfactant requirement (12, 13). Our team weighted the scores of SIMPLE criteria according to the previously defined PDA risk factors and also their prior subjective clinical experience as our NICU is a tertiary reference center caring for ~70 ELBW infants per year, and some risk factors have already been identified for hsPDA in routine clinical follow-up of these infants. Clinical chorioamnionitis was defined as the presence of fever, uterine fundal tenderness, maternal tachycardia (>100/min), fetal tachycardia (>160/min), and purulent or foul amniotic fluid (14). All these parameters were scored between 0 and 3 points as shown in Table 1, and the maximum score is 29. The scores for all patients were recorded at postnatal 6, 12, 18, 24, 48, and at 72 h of life.

**Table 1:** Criteria of SIMPLE scoring system.

	**0**	**1**	**2**	**3**
Presence of maternal chorioamnionitis	No	Yes		
Completed cure of antenatal steroid	Yes	No		
Birth weight, (g)	>1,250	1,000–1,250	750–1,000	<750
Cord blood gas base deficit (mmol/L)	<−12	−12 to −16	>−16	
*Presence of hypotension requiring inotropic support	No	Yes		
*Presence of tachycardia (beat/min)	<160	160–180	>180	
*Need of invasive MV	no	yes		HFOV
*^#^Presence of metabolic acidosis	pH: >7.35	7.25–7.35	7.10–7.25	<7.1
	BD: < −4	−4 to −10	−10 to −16	< −16
*Presence of respiratory acidosis (PCO_2_, mmHg)	35–45	45–55	55–65	>65
*Maximum PIP (cmH_2_O)	<18	18–23	>23	
PIP reduction % according to prior assessment	>20%	%10–20	<10%	
FiO_2_ at admission (%)	<40	40–60	>60	
FiO_2_ reduction % according to prior assessment	>20%	10%−20%	<10%	
Number of surfactant administered	0	1	>1	

* *During evaluation episode*.

# *The highest score will be given according to pH or BD*.

*BD, base deficit; HFOV, high frequency oscillatory ventilation; MV, mechanical ventilation; PIP, peak inspiratory pressure*.

The antenatal characteristics of infants, presence of maternal chorioamnionitis, neonatal demographical data, cord blood gas analyses, clinical findings, and neonatal morbidities including neonatal sepsis, NEC, IVH, ROP, BPD, and mortality were all recorded.

Gestational age of the infants was determined according to the last menstrual period of the mother and antenatal ultrasonographic evaluation. Turkish Neonatology Society (TNS) guideline was used for the diagnosis and treatment of respiratory distress syndrome (RDS) (15). BPD was determined according to 2001 National Institute of Child Health and Human Development (NICHD) consensus statement as requirement for supplemental oxygen for the first 28 days of life and was classified into three groups in terms of BPD severity (mild, moderate, and severe), depending on the duration and level of supplemental oxygen and mechanical ventilatory support at 36-week postmenstrual age (16). NEC was diagnosed considering clinical and radiographic findings and was classified according to the modified Bell's criteria (17). The Volpe classification was used for the IVH staging (18). ROP was classified according to The International Classification of Retinopathy of Prematurity (19). Neonatal sepsis was diagnosed according to the criteria of TNS neonatal infection diagnosis and treatment guideline (20).

The echocardiographic evaluations of all infants were performed at postnatal 24, 48, and 72 h of life by the same pediatric cardiologist. The Vivid E BT12 ultrasonography device Vivid E BT12 (General Electrics, MA, US) was used for the echo evaluation. The ultrasonographical evaluation was performed as two-dimensional imaging, M-mode, color flow mapping, diastolic retrograde flow measurement, and Doppler imaging. The ratio of inner diameter of DA to infant weight and the ratio of left atrium to aorta (LA/Ao) were recorded. The criteria for hsPDA were either being the inner diameter of DA > 1.5 mm/kg and/or LA/Ao > 1.5 according to the TNS PDA guideline in preterm infants (21). A medical PDA closure treatment was initiated for infants with hsPDA who had accompanying symptoms including respiratory compromise (requiring persistent mechanical support, increased ventilatory support, and increased oxygen demand), heart failure, metabolic acidosis or large left-to-right ductus shunt with evidence of hemodynamic compromise, such as reversal of flow in the descending aorta during diastole, oliguria or rising serum creatinine concentration, hypotension, or widened pulse pressure (1). Either ibuprofen or paracetamol was given according to clinical findings of the infants and laboratory data. Ibuprofen was used for three consecutive days at a dose of 10 mg/kg/day on the 1st day, 5 mg/kg/day on the 2nd day, and 5 mg/kg/day on the 3rd day (21). The paracetamol treatment was administered for three consecutive days at a dose of 15 mg/kg q6h in the presence of contraindications for ibuprofen therapy, such as thrombocytopenia (<60.000/mm^3^), active bleeding (IVH and gastrointestinal hemorrhage), acute renal failure, or NEC (21).

Statistical analysis was performed by IBM SPSS 22.0 (IBM SPSS for Windows version 22, Armonk, NY, USA). Absolute and relative frequencies were determined for nominal scaled parameters. The consistency of continuous variables to normal distribution was tested using the Shapiro–Wilk test. Descriptive statistics are given as median (range) for continuous variables and *n* (%) for categorical variables. The Mann–Whitney *U*, Pearson's chi-square, and Fisher exact tests were used for appropriate comparisons between groups. To compare the quality of combinations of specific features, receiver operating characteristic (ROC) curves were used to determine the sensitivity and specificity by calculating the area under the curve (depending on the specified cut-off value). The ROC analysis was performed using the MedCalc for Windows, version 19.4 (MedCalc Software, Ostend, Belgium). The ROC curves, cut-off points, and area under curve (AUC) were all compared with each other using the MedCalc statistical software (Ostend, Belgium). A *p* < 0.05 was considered statistically significant.

## Results

A total of 48 ELBW infants were included in the study. Within the subjects, 26 (54%) were male. The median gestational age and birth weight of infants were 25 weeks and 730 g, respectively. Thirty-four (70%) infants were intubated at the time of admission to NICU, and the median duration for invasive ventilatory support and oxygen treatment were 13 and 31 days, respectively. The median length of stay at hospital was 52 days (Table 2).

**Table 2:** The demographic data of all infants.

**Parameters**	**All infants (*n* = 48)**	**Infants with hsPDA (*n* = 30)**	**Infants without PDA (*n* = 18)**	***p-*value**
Gender (male/female, *n*)	26/22	16/14	10/8	0.34
Gestational age; weeks median (range)	25 (21–29)	25 (21–29)	26 (24–29)	0.06
Birth weight; grams median (range)	730 (340–995)	720 (340–990)	760 (620–995)	0.06
Duration of invasive MV; days median (range)	13 (0–69)	17 (3–69)	9 (0–62)	0.11
Duration of oxygen treatment; days median (range)	31 (2–106)	47 (9–106)	34 (2–69)	0.23
Length of stay at hospital; days median (range)	52 (3–151)	58 (3–142)	43 (34–151)	0.39

*hsPDA, hemodynamic significant PDA; MV, mechanical ventilation*.

A total of 30 (62.5%) infants required medical PDA closure treatment. The median gestational age and birth weight of the infants with hsPDA were 25 weeks and 720 g, whereas the median gestational age and birth weight of infants without PDA were 26 weeks and 760 g, respectively; the difference was not statistically significant. There were no significant differences between infants with and without hsPDA in terms of duration of MV, oxygen treatment, and hospitalization (Table 2). The presence of maternal chorioamnionitis, lack of antenatal steroid administration, hypotension, tachycardia, oliguria, and recurrent surfactant requirement and need of high-frequency oscillatory ventilation were all found to be higher in the hsPDA group compared to the no-PDA group (Table 3). Both groups were similar in terms of NEC, BPD, late sepsis, and ROP development. The IVH rate was significantly higher in the hsPDA group. A total of 16 (33%) infants died, all of whom had hsPDA (Table 4).

**Table 3:** Subject characteristics and SIMPLE score items.

**Parameters**	**Infants with hsPDA (*n* = 30)**	**Infants without PDA (*n* = 18)**	***p*-value**
Gestational age, weeks, median (range)	25 (21–29)	26 (24–29)	0.06
Birth weight, g, median (range)	720 (340–990)	760 (620–995)	0.06
Maternal chorioamnionitis, *n* (%)	21 (70)	5 (28)	0.005
Lack of antenatal steroid therapy, *n* (%)	19 (63)	4 (22)	0.006
Hypotension requiring inotropic support, *n* (%)	22 (73)	2 (11)	<0.001
Tachycardia, *n* (%)	16 (53)	3 (17)	0.01
Oliguria, *n* (%)	11 (37)	1 (5.5)	0.01
Surfactant treatment, *n* (%)	16 (53)	6 (33)	0.14
Repeated dosage of surfactant (%)	9 (30)	0 (0)	0.001
High frequency ventilation, *n* (%)	9 (30)	1 (5)	0.003
Cord blood gas deficit ≥−12 mmol/L, *n* (%)	7 (14.6)	4 (8.3)	0.92
Need of invasive ventilation, *n* (%)	26 (54.2)	15 (31.3)	0.75
Metabolic acidosis, pH < 7.35, BD > −4, *n* (%)	7 (14.6)	4 (8.3)	0.92
Respiratory acidosis, pH < 7.35, PCO_2_ > 45 mmHg, *n* (%)	26 (54.2)	15 (31.3)	0.75
Maximum PIP, >18 cm H_2_O, *n* (%)	22 (18–24)	22 (18–24)	0.62
FiO_2_ at admission, >40%, *n* (%)	26 (54.2)	15 (31.3)	0.75

**Table 4:** Mortality and co-morbidities between study groups.

**Parameters**	**Infants with hsPDA (*n* = 30)**	**Infants without PDA (*n* = 18)**	***p*-value**
IVH ≥ Grade 3, 3rd day, *n* (%)	15 (50)	1 (6)	<0.001
IVH ≥ Grade 3, 7th day, *n* (%)	17 (57)	1 (6)	<0.001
NEC ≥ stage II, *n* (%)	7 (23)	2 (11)	0.31
BPD (moderate-severe), *n* (%)	12 (40)	7 (39)	0.53
Late sepsis, *n* (%)	12 (40)	12 (67)	0.08
ROP, *n* (%)	19 (63)	13 (72)	0.26
Mortality, *n* (%)	16 (53)	0	<0.001

*BPD, bronchopulmonary dysplasia; IVH, intraventricular hemorrhage; NEC, necrotizing enterocolitis; ROP, retinopathy of prematurity*.

The median LA/Ao ratios of all infants were 1.57, 1.55, and 1.5 and the PDA diameter/infant weight ratios were 2.02, 1.9, and 1.9 at 24, 48, and 72 h of life, respectively. The median LA/Ao ratio was significantly higher in the PDA treatment group at 24, 48, and 72 h of life; the median PDA diameter/infant weight ratio was significantly higher in the treatment group at 48 and 72 h of life (Table 5).

**Table 5:** Echocardiography indices of the study population.

**Parameters**	**Infants with hsPDA (*n* = 30)**	**Infants without PDA (*n* = 18)**	***p*-value**
La/Ao ratio, day 1 median (range)	1.7 (1.2–2.8)	1.3 (1–2.3)	0.009
La/Ao ratio, day 2 median (range)	1.6 (1.3–3.4)	1.2 (0.9–1.8)	<0.001
La/Ao ratio, day 3 median (range)	1.6 (1.2–2.9)	1.2(0.9–1.5)	<0.001
PDA diameter/weight, day 1 median (range)	2.1 (1.1–3.7)	1.9 (1.2–2.7)	0.27
PDA diameter/weight, day 2 median (range)	2.1 (1.1–3.8)	1.6 (1.3–2.3)	<0.001
PDA diameter/weight, day 3 median (range)	2.3 (0.9–3.5)	1.3 (1.1–2.1)	<0.001

*La, left atrium; Ao, aorta*.

During NICU hospitalization, 30 infants with symptomatic hsPDA (62%) required intervention. The PDA closure treatments were initiated at 24, 48, and 72 h of life in 13 (43%), 14 (47%), and 3 (10%) infants, respectively. The ibuprofen treatment was initiated in 5 (16%), infants and the paracetamol treatment was initiated in 25 (84%) infants. The Follow-up echocardiographic evaluation showed closure of PDA immediately after the first dose in 26 (86%) infants, whereas 4 (14%) infants received repeated doses of the medical treatment. A surgical ligation was performed in three (10%) infants. All cases had permanent closure and no infants revealed symptoms of reopening after effective closure of PDA.

**Figure 2:**
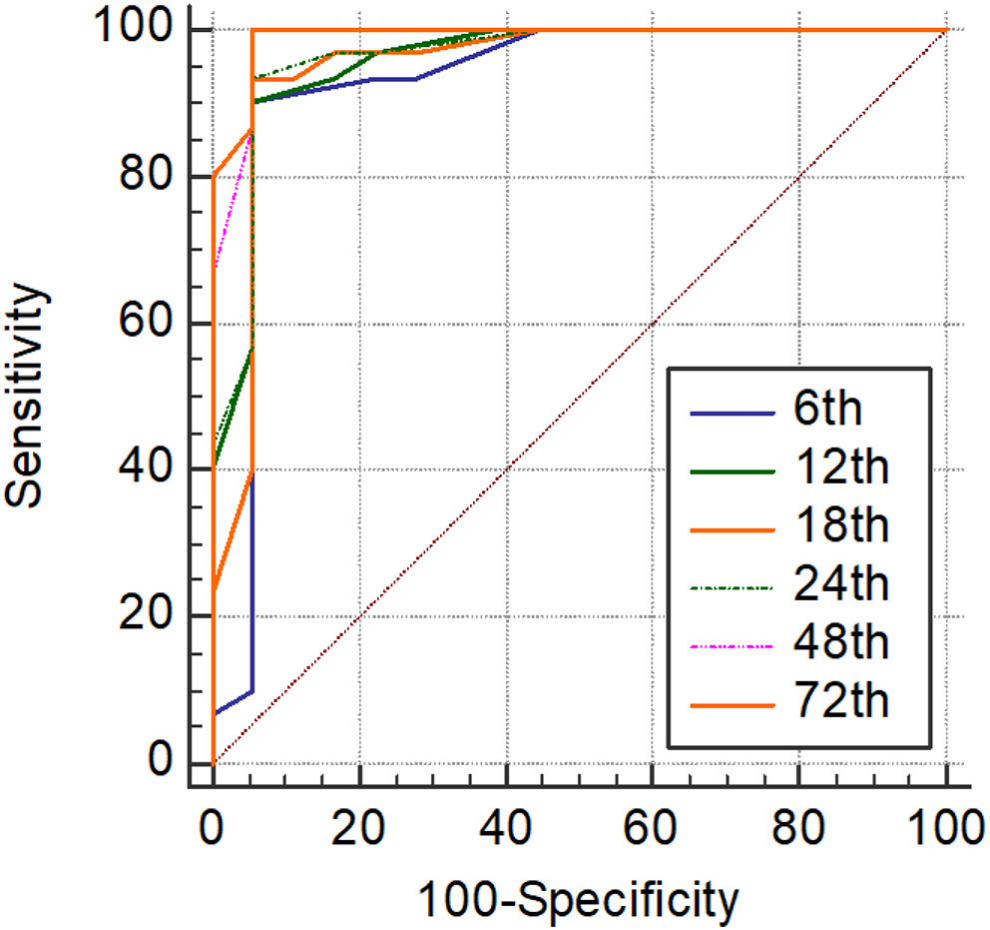
Receiver operating characteristics (ROC) analyses of SIMPLE scoring system at each time point.

Significantly higher scores were obtained by using SIMPLE in infants with hsPDA compared with those without PDA (*p* < 0.05) (Table 6). Hypotension, tachycardia, oliguria, surfactant administration, and high frequency ventilation were significantly higher in the hsPDA group. The SIMPLE scores were similar between two groups of infants who had either medical or surgical closure. The ROC statistics demonstrated a high sensitivity and specificity of the SIMPLE scores, which did not significantly differ between different time points from 6 to 72 h. The cut-off SIMPLE scores that were found to be statistically significant for the detection of symptomatic hsPDA for each time were identified (Table 7, Figure 2). However, AUC for established cut-off values did not statistically differ between different time points (data not shown).

**Table 6:** SIMPLE scores in infants with/without hemodynamically significant patent ductus arteriosus (hsPDA) by echocardiography.

**Evaluation time**	**SIMPLE scores in infants with hsPDA (*n* = 30) median (range)**	**SIMPLE scores in infants without PDA (*n* = 18) median (range)**	***p*-value**
6 h	12 (5–19)	4 (2–18)	<0.001
12 h	13 (5–20)	4 (2–13)	0.001
18 h	13 (5–22)	4 (2–14)	<0.001
24 h	14 (5–19)	4 (2–14)	<0.001
48 h	15 (10–23)	4 (2–13)	<0.001
72 h	14 (9–21)	4 (2–12)	<0.001

**Table 7:** Receiver operating characteristics (ROC) curve statistics for SIMPLE scores at various time points 6–72 h.

**Time point**	**Sensitivity (95% CI)**	**Specificity (95% CI)**	**Area under curve (AUC)**	**Cut-off value**	*p*-value*
6 h	92.00 (73.5–98.7)	85.00 (67.7–92.6)	0.926	>8.5	<0.001
12 h	90.00 (73.5–97.9)	94.44 (72.7–99.9)	0.956	>8	<0.001
18 h	93.33 (77.9–99.2)	94.44 (72.7–99.9)	0.949	>8	<0.001
24 h	93.33 (77.9–99.2)	94.44 (72.7–99.9)	0.961	>8	<0.001
48 h	100.00 (88.4–100.0)	94.44 (72.7–99.9)	0.987	>6	<0.001
72 h	100.00 (88.4–100.0)	94.44 (72.7–99.9)	0.991	>6	<0.001

* *Each p-value shows the statistical significance of AUC for the given time point*.

## Discussion

The utility of a novel scoring system that can be easily applied during the first 3 days of life for prediction of hsPDA in ELBW preterm infants was investigated in this prospective study. The main advantage of SIMPLE consists of the fact that it does not require any echo evaluation. The scoring systems consists of 14 parameters that are related with several risk factors associated with the hsPDA development. SIMPLE was found to predict hsPDA requiring medical intervention from 6 h of life with high specificity and sensitivity. It may also be used as a screening tool to establish the requirement for echo and may prevent unnecessary echo evaluation in NICUs.

The main antenatal risk factors for PDA include maternal chorioamnionitis, maternal pregnancy-induced hypertension, and lack of antenatal steroid administration (1–3, 22). Maternal chorioamnionitis was shown to increase the risk of PDA by 1.43 times (12). Similarly, maternal chorioamnionitis was significantly higher in the hsPDA group in our study. Therefore, chorioamnionitis may be suggested as an important criterion of SIMPLE for early prediction of hsPDA in the ELBW infants. Antenatal steroids had an impact on the incidence of PDA in infants ≤34 weeks of gestational age, whereas its effect in the smallest infants are still conflicting (23). The antenatal steroid treatment reduced the risk of symptomatic PDA (22). Therefore, the antenatal steroid administration status was also included to SIMPLE. As 83% of infants with hsPDA had not received antenatal steroids, we suggest that the lack of antenatal steroid administration is an important risk factor for hsPDA in the ELBW infants. In addition, the requirement of the PDA treatment was significantly lower in infants born to mothers without chorioamnionitis (24). All these data may support the idea of implementation of two important antenatal risk factors in SIMPLE. Also, postnatal risk factors, including prematurity, low birth weight, and gender, were all suggested to be important for the development of PDA in neonates (25). Perinatal asphyxia, respiratory distress, surfactant usage, fluid overload, and sepsis represent common postnatal risk factors of PDA (1–3, 22). The incidence of PDA increases as the gestational age decreases, and 60–70% of infants <28 weeks of gestation receive medical or surgical closure treatment (26). Consistent with the literature, PDA was diagnosed in 95, 93, and 85% of ELBW infants at 24, 48, and 72 h of life, respectively (27). In addition, surfactant use and need for re-dosing were also strongly associated with hsPDA, as previously published (28). Therefore, SIMPLE was found to predictive of hsPDA by including both antenatal and postnatal risk factors.

The most common findings of PDA include widened pulse pressure, systolic murmur, tachycardia, hypotension, pulmonary edema, increased mechanical ventilatory and/or oxygen support, pulmonary hemorrhage, metabolic acidosis, oliguria, and feeding intolerance (5, 10, 25). Similarly, hypotension, tachycardia, oliguria, and requirement of high frequency ventilation were significantly higher in the hsPDA group in our study. Therefore, we suggest that SIMPLE, including common PDA findings in postnatal period, may be used to determine hsPDA. The evaluation of clinical findings objectively from the 1st h of life in a non-invasive manner may offer an important advantage for SIMPLE for early prediction of hsPDA in ELBW infants.

Echocardiography represents the most widespread diagnostic technique, allowing for measurement valuation of the PDA diameter and Doppler flow direction (29). Early ductal diameter of >1.5 mm/kg was found to predict symptomatic PDA with high sensitivity (30). Since significant shunt through PDA may remain clinically “silent” in the 1st days and the development of echocardiographic signs of hsPDA may precede the development of clinical signs by a mean of 2 days, an early selective PDA screening by echo has been recommended in ELBW infants (21, 31, 32). The early echo screening was also shown to be associated with lower in-hospital mortality and likelihood of pulmonary hemorrhage in ELBW infants (33). Therefore, all infants were screened daily with echo in the first 3 days of life in our study to evaluate the efficacy of SIMPLE. However, it may not always be possible to perform echo in every NICU. In contrast to the previous PDA scoring systems, we did not include echo findings in SIMPLE for the purpose of providing an echo -independent scoring system. As minimal handling and maximal comfort are important for the quality of ELBW care, we sought to keep the infants warm, stable, and free from infection by identifying those with hsPDA while avoiding unnecessary, repeated echo examinations (34). In addition, although ELBW infants usually require serial echo evaluation, the number of pediatric cardiologists are not enough to perform all of them at every time point in most of NICUs (35). The SIMPLE scores of infants with hsPDA were higher at all time points. In addition, ELBW infants who received the PDA treatment had also significantly higher SIMPLE scores. Therefore, we suggest that SIMPLE may be used as a time- and cost-effective screening tool in ELBW infants who need echo for the confirmation of hsPDA and for the requirement of medical PDA closure. In this context, SIMPLE may prevent unnecessary echo evaluations in ELBW infants and increase the comfort of the infant. It can also guide neonatologists to predict hsPDA without echo findings and may offer an advantage to perform selective echo in ELBW infants in the presence of higher scores, especially in NICUs where it is not possible to perform echo at any time. As studies, such as PDA-Tolerate and INTERPDA which evaluated conservative management in PDA, suggested a new approach for the late echocardiographic evaluation, further randomized controlled studies are required to determine the ideal time of echo screening in ELBW infants (36, 37).

Patent ductus arteriosus is known to be associated with important neonatal morbidities (9, 10). The potential consequences of hsPDA include increased risk for prolonged ventilation, BPD, NEC, IVH, pulmonary hemorrhage, and death (6). However, it is also well-known that all of these are mainly prematurity-associated late morbidities with multifactorial pathogenesis. NEC, BPD, late sepsis, and ROP rates were similar in infants with and without hsPDA in our study. This similarity therefore may suggest validation of SIMPLE for early and accurate prediction of hsPDA in ELBW infants. IVH and mortality rates were significantly higher in the PDA treatment group in this study. As PDA requiring treatment was found to be associated with a higher risk of IVH in a recent study. IVH may be a consequence of hsPDA (38). This finding is in accordance with a previous report indicating an increased risk of death by 3.4 times in the presence of PDA on the 3rd day in ELBW infants (39). In the light of these data, we suggest that hsPDA may be an important contributor for both IVH and mortality in ELBW infants, in spite of medical treatment for closure.

Several severity scores were established for the prediction of PDA depending on the echo findings and seem to be more subjective and usually applied after 2 days of life (7–11, 40). One of them, mainly based on clinical parameters, such as respiratory worsening, acidosis, increased femoral pulses, and precordium pulsatility, from the 2nd to the 10th day of life, reached the highest efficacy and specificity on day 4 (10). However, as this scoring system requires two different physical examination criteria, scoring might be subjective in case two different physicians perform physical examinations. Also, the administration of the scoring from the 2nd day of life might be associated with failure of early prediction of PDA. Another PDA scoring system included findings, such as heart rate, murmur, peripheral pulses, precordial pulsation, and cardiothoracic index. However, it is applied initially on the 4th day of life, and it has also to be confirmed with echo (7). The main advantage of SIMPLE was to be performed by objective evaluation of 14 clinical parameters without any dependence of echo compared with the previous scoring systems. SIMPLE scores from 6th h of life at each time point were all higher in infants with hsPDA who required medical intervention. In addition, cut-off scores at each time points for the detection of hsPDA requiring the PDA closure treatment were also established. In SIMPLE, echo might be used for the confirmation of hsPDA diagnosis. As a result, we suggest to use SIMPLE to predict infants who may develop hsPDA from early hours of life and perform the echo evaluation in a more targeted and selective way to confirm PDA diagnosis. This strategy has the potential to decrease unnecessary echo evaluation and maximize the comfort of the ELBW infant. The physician who follows-up the high risk infants for findings of hsPDA may be able to start PDA closure treatment in the most appropriate time period. In addition, in NICUs with limited ability to perform echo at any time, SIMPLE may suggest the neonatologist to identify the ELBW infants for hsPDA and consult with a pediatric cardiologist on the most suitable time period and/or make decision for the medical closure of PDA. However, SIMPLE scores could not predict the response to the PDA closure treatment as they were similar in both medical closure and surgical ligation groups. This may be associated with small number of infants who required surgical ligation.

On the other hand, the present study is limited with the low number of infants enrolled, the single NICU involved in the study and evaluation of the efficacy of SIMPLE only by identification of high-risk infants. We believe that the efficacy of SIMPLE for prediction of long-term outcomes and PDA-associated morbidities, such as NEC, IVH, ROP, and mortality, should also be investigated. Therefore, we aim to evaluate the efficacy of SIMPLE for early prediction of PDA, morbidities associated with PDA, and also mortality in ELBW infants in a larger multicentered prospective observational study. Another limitation of this study is that the incidence of PDA and several comorbidities are higher than in other Western cohorts and registries such as Vermont Oxford Network or NICHD. This is likely to be related to the very low prevalence of antenatal steroid treatment compared to the US (41). This may be due to the fact that our maternal population is mostly underserved, including many migrants with limited or no prenatal care. Thus, SIMPLE should be validated in other populations with standard prenatal care.

In conclusion, to the best of our knowledge, SIMPLE is the first scoring system that relies only on risk factors and clinical findings for the early prediction of hsPDA in ELBW infants. It is simple, objective, and easy to perform. It does not require any additional tests and/or echo during the evaluation. Therefore, we suggest that it can be used as a screening tool for the identification of ELBW infants for their requirement for the echo evaluation. In addition, SIMPLE will probably minimize the unnecessary pediatric cardiology consultations and/or echo evaluation in NICUs with limited sources. It may also lead to early diagnosis and treatment of hsPDA in ELBW infants as it can be performed within the first 24 h of life. However, its efficacy on both early identification of hsPDA- and PDA-associated morbidities, and also mortality should be evaluated in a multicentered prospective study including higher number of premature infants.

## Data Availability Statement

The original contributions presented in the study are included in the article/supplementary material, further inquiries can be directed to the corresponding author/s.

## Ethics Statement

The studies involving human participants were reviewed and approved by Kanuni Sultan Suleyman Training and Research Hospital Local Ethical Committee. Written informed consent to participate in this study was provided by the participants' legal guardian/next of kin.

## Author Contributions

IG and AB conceptualized and designed the study, collected, analyzed, interpreted data, and performed the literature search. HB performed echocardiographic evaluations of infants. BY performed the literature search, carried out initial analyses, interpreted data, and helped to draft article. SM and SS performed literature search and interpreted data. MC designed the study, analyzed the data, and wrote the manuscript. All authors contributed to the article and approved the submitted version.

## Conflict of Interest

The authors declare that the research was conducted in the absence of any commercial or financial relationships that could be construed as a potential conflict of interest.
